# Potential of Indigenous Strains Isolated from the Wastewater Treatment Plant of a Crude Oil Refinery

**DOI:** 10.3390/microorganisms11030752

**Published:** 2023-03-15

**Authors:** Signe Viggor, Merike Jõesaar, Celeste Peterson, Riho Teras, Maia Kivisaar

**Affiliations:** 1 Institute of Molecular and Cell Biology, University of Tartu, 23 Riia Street, 51010 Tartu, Estonia; 2 Institute of Biomedicine and Translational Medicine, University of Tartu, 19 Ravila Street, 50411 Tartu, Estonia

**Keywords:** *Acinetobacter*, *Pseudomonas*, alkane hydroxylases, hydrophobicity, crude oil

## Abstract

Contamination of the environment with crude oil or other fuels is an enormous disaster for all organisms. The microbial communities for bioremediation have been an effective tool for eliminating pollution. This study aimed to determine individual cultures’ and a strain mixture’s ability to utilize alkanes (single alkanes and crude oil). The proper study of pure cultures is necessary to design synergistically working consortia. The *Acinetobacter venetianus* ICP1 and *Pseudomonas oleovorans* ICTN13 strains isolated from a wastewater treatment plant of a crude oil refinery can grow in media containing various aromatic and aliphatic hydrocarbons. The genome of the strain ICP1 contains four genes encoding alkane hydroxylases, whose transcription depended on the length of the alkane in the media. We observed that the hydrophobic cells of the strain ICP1 adhered to hydrophobic substrates, and their biofilm formation increased the bioavailability and biodegradation of the hydrocarbons. Although strain ICTN13 also has one alkane hydroxylase-encoding gene, the growth of the strain in a minimal medium containing alkanes was weak. Importantly, the growth of the mixture of strains in the crude oil-containing medium was enhanced compared with that of the single strains, probably due to the specialization in the degradation of different hydrocarbon classes and co-production of biosurfactants.

## 1. Introduction

The processing of crude oil and the use of the products made from it are often accompanied by environmental pollution. The damage caused by these complex and toxic compounds is usually devastating to the whole ecosystem. One way to deal with this kind of pollution is by using microbes possessing versatile metabolic potentials for the degradation of aromatic and aliphatic hydrocarbons, the main components of crude oil. Particular attention should be paid to strains capable of degrading both types of compounds [1].

Crude oil refinery wastewater treatment plants (WWTP) are hotspots for microorganisms specialized for degrading different pollutants. Isolation and characterization of such microbes allow us to find the most effective strains for eliminating pollution with bioremediation techniques [1,2,3]. Microbial communities play a significant role in the metabolism of organic matter, and therefore it is essential to analyze the dynamics of the communities responsible for pollutant degradation [4]. To achieve fast and effective degradation, a community member may provide carbon sources for others, degrade toxic metabolites/intermediates that otherwise accumulate in the growth medium, etc. [5]. Therefore, it is necessary to know the physiology of individual strains to construct an efficient working community.

Alkanes are the primary pollutants in oil-contaminated environments as they constitute up to 50% of crude oil, depending on the source [6]. At the same time, many organisms such as bacteria, green algae, plants, or animals produce alkanes; therefore, alkanes are present in soil and water in small amounts [7,8,9]. Thus, due to the presence of alkanes in natural environments, alkane-degradation enzymes are common among bacteria. There are several enzymes for the aerobic degradation of alkanes in bacteria: soluble cytochrome P450-related (CYP153 family) for C_5_–C_16_, AlkB-type enzymes for C_3_–C_20_, and AlmA and LadA for C_20_–C_32_ and C_15_–C_36_ alkanes, respectively [6,10,11]. AlkB is the most studied alkane hydroxylase in Gram-negative and Gram-positive bacteria. It is an integral membrane non-heme di-iron monooxygenase and requires rubredoxin and rubredoxin reductase for electron transfer during the degradation of alkanes [9,12]. Microorganisms may have five or more alkane degradation systems, each one being active on alkanes of a certain chain length or being expressed under specific physiological conditions [12]. For example, *Alcanivorax borkumensis* has two AlkB-like alkane hydroxylases and three cytochrome-P450-encoding genes [13]. Thus, alkane hydroxylase encoding genes are also good markers to predict the potential of bacteria for oil degradation in the case of oil pollution [14].

Bacteria secrete biopolymers, for example, biosurfactants [9,15,16], to enhance hydrophobic hydrocarbon uptake and thus their degradation [17,18]. Biosurfactants also play an important role in microbial adhesion to and desorption from surfaces or biofilms [19]. The formation of biofilms is a complex process that depends on different cellular and environmental factors such as the hydrophobicity of cells, availability of nutrients, temperature, charge and roughness of the surface, etc. [20]. It has been suggested that biofilm-forming bacteria have an advantage over planktonic bacteria as improved cell survival, metabolism, adaptation, and propagation are provided by the biofilm matrix [21]. For example, a positive correlation has been found between biofilm formation and alkane degradation of *Acinetobacter oleivorans* DR1 [22].

Research focusing on microbial cell properties, pollutant degradation, surfactant production, and biofilm formation under various environmental conditions is the basis for the development of pollution remediation applications.

The current study explores two indigenous bacterial strains, *Acinetobacter venetianus* ICP1 and *Pseudomonas oleovorans* ICTN13, isolated from the WWTP of an Indian crude oil refinery [2]. Our previous experiments revealed that these strains could grow on aromatic (phenol, cresols, toluene) and aliphatic (hexadecane) hydrocarbon-containing minimal agar plates and possess phenol degradation operons. Additionally, structurally mosaic operon-carrying genes related to a toluene catabolic operon was identified in the genome of ICTN13 [2].

To further elucidate the genetic basis of the broad catabolic potential of these strains, the current study focused on identifying the genes responsible for the degradation of aliphatic hydrocarbons. Our results revealed that the strain ICP1 contains four alkane hydroxylase genes, and the strain ICTN13 has one gene encoding an alkane hydroxylase. Improved degradation of alkanes was observed with the mixture of the strains ICP1 and ICTN13.

## 2. Materials and Methods

### 2.1. Strains Used in the Study

*Acinetobacter venetianus* ICP1 and *Pseudomonas oleovorans* ICTN13 were isolated from plated cultures of samples collected from an Indian crude oil refinery wastewater treatment plant (WWTP) clarifier outlet in January 2016 on selective M9 minimal agar plates containing phenol (1.3 mM, Sigma-Aldrich, St. Louis, MO, USA; 99% purity) or crude oil (CO; PJSC Lukoil Oil Company, Moscow, Russia) as the only growth substrate [2]. The information concerning the sequencing and analysis of the genomes of the strains is available in Viggor et al., 2020 [2].

### 2.2. Growth on Minimal Medium Containing Phenol or/and Hexadecane

The strains ICTN13 and ICP1 were pre-grown overnight in an R2A medium containing 1.3 mM phenol or 0.1% hexadecane (30 °C; 130 rpm). The cells were separated using centrifugation (5000 rpm, 10 min) and resuspended in an M9 minimal medium. The growth of the ICP1, ICTN13, and mixture of the strains on M9 minimal medium containing 1.3 mM phenol, 0.1% hexadecane, or both substrates was followed spectrophotometrically at 580 nm.

### 2.3. Growth on Minimal Medium Supplemented with 1% Crude Oil

The ICP1 and ICTN13 strains were grown overnight on the R2A medium supplemented with 1% hexadecane (Sigma-Aldrich, St. Louis, MO, USA; 99% purity) at 30 °C. The cells were separated using centrifugation (5000 rpm, 10 min), resuspended in the minimal medium, and used as inoculum for a minimal medium containing 1% crude oil. Identical flasks without cells, were prepared for abiotic controls. Erlenmeyer flasks (250 mL) containing 50 mL growth medium were incubated on a rotary shaker (100 rpm). The growth of strains was determined spectrophotometrically at 580 nm. At each time point, separate flasks were sacrificed for the chemical analyses. Three independent experiments were conducted.

### 2.4. Chemical Analysis of Growth Media

Samples for the determination of crude oil degradation efficiency were taken after inoculation and after 24 h and 5 days of incubation from abiotic controls and microcosms. All samples were stored at −20 °C until the analysis. Crude oil hydrocarbons were extracted with dichloromethane [23]. Combined extracts of the sample were centrifuged (5000 rpm for 10 min) to separate water residues and were later analyzed by a GC-MS system consisting of an Agilent 6890N Network GC System (Agilent Technologies, Santa Clara, CA, USA) and Agilent 5973N Mass Selective Detector (Agilent Technologies, Santa Clara, CA, USA) operating in electron ionization mode. The capillary column DB-5ms, 30 m in length with an internal diameter of 0.25 mm and stationary-phase film thickness of 0.25 μm (Agilent Technologies, Santa Clara, CA, USA), was used for the separation of compounds. Helium was used as a carrier gas at a 0.7 mL/min flow rate. The injection volume was 1 μL, injector temperature and split ratio were 280 °C and 25:1, respectively. The initial oven temperature was 70 °C, isothermal hold lasted for 5 min, which was then increased at 3 °C/min to 310 °C with the 5 min hold, with an additional 5 min at 310 °C for column cleaning. The total run time was 90 min. The mass spectrometer was operated in the scan mode of 50–550 *m*/*z* mass range, solvent delay 3 min, and electron ionization with 70 eV electrons was used. The chromatograms were obtained and analyzed with Agilent MSD ChemStation E.02 and with the NIST Mass Spectral Search Program 2.2 and database (2014). The results of one growth experiment are presented. The relative concentration of n-alkanes was calculated by the ratio of the peak area of the compound in the sample to the peak area of the same compound in 0 h abiotic control.

### 2.5. qRT-PCR and Growth on R2A Medium Supplemented with 0.2% Substrate

*A. venetianus* strain ICP1 was grown in 250 mL Erlenmeyer flasks containing 50 mL of R2A medium, R2A supplied with–0.2% nonane (C_9_H_20_), dodecane (C_12_H_26_), tetradecane (C_14_H_30_), hexadecane (C_16_H_34_), octadecane (C_18_H_38_), docosane (C_22_H_50_), dotriacontane (C_32_H_66_), squalane (S; 2,6,10,15,19,23-hexamethyltetracosane; C_30_H_62_), or crude oil (CO). All alkanes (99% purity) were purchased from Sigma-Aldrich (St. Louis, MO, USA). RNA was isolated from 1 mL samples taken from different phases of the growth curve (cells were spun for 3 min, 13,000 rpm) to assess the expression of *alkMa*, *alkMb*, *alma*, and *P450* genes. A Nucleospin^®^ RNA Kit (Machery-Nagel GmbH & Co., Dueren, Germany) was used to isolate RNA according to the manufacturer’s protocol. Isolated RNA samples were subjected to DNase treatment by using the enzyme DNase I (Thermo Fischer Scientific, Waltham, MA, USA; 37 °C for 30 min) and followed by precipitation with 3 M sodium acetate (pH 5.1) and 96% ethanol at −20 ° C for 1 h. A NanoDrop ND-1000 spectrophotometer (Thermo Fisher Scientific, Waltham, MA, USA) was used to determine the concentration and purity of the isolated RNA.

The qRT-PCR assays were carried out with the SYBR Green 1-Step qPCR Low Rox Kit (Thermo Fisher Scientific, Waltham, MA, USA) in a Rotor-Gene Q (Qiagen, Hilden, Germany) using the following program: cDNA synthesis 50 °C for 15 min; denaturing at 95 °C for 15 min; 40 cycles of denaturing 95 °C for 15 s and annealing at 60 °C for 30 s; and elongation at 72 °C for 30 s, followed by a melting curve from 72 to 95 °C. Each qRT-PCR reaction mixture contained 10 ng of isolated RNA. The samples were amplified using the primers listed in Table S1. The *rpoB* (RNA polymerase beta subunit) gene was used as a reference gene (Table S1). The samples were amplified in triplicate and under the same conditions. The relative levels of the tested genes were calculated by LinReqPCR (2020.2) [24]. One-way ANOVA (α = 0.05) was used for statistical analysis of the data, and analysis was performed with TIBCO Statistica v. 13.5.0.17 (TIBCO Software Inc., Palo Alto, CA USA). Spearman Rank Order Correlations (RT_substr) conditions: MD pairwise deleted; marked correlations were considered significant at *p* < 0.05; and include condition: v1 = ‘gene’ and v2 = 0.

### 2.6. MATH Test

Cells grown overnight on 1.3 mM phenol or hexadecane-supplemented R2A medium were collected by centrifugation (12 min 5000 rpm). The obtained pellet was washed twice with PUM buffer [25] and resuspended in the same medium so that the A_580_ of the solution was approximately 0.4. To determine the hydrophobicity of the cell surface, the MATH (Microbial Adherence to Hydrocarbons) test was conducted with some modifications of the previously described method by Rosenberg et al. (1980) [25]. Briefly, 1.2 mL of cell suspension was pipetted into a 2 mL Eppendorf tube, and 0.12 mL of hexadecane, octane, or p-xylene was added. The tubes were mixed on a shaker for 2 min. After 15 min of rest, the absorbance of the aqueous phase was measured. The adhesion of bacteria to the solvents was evaluated by applying the following formula: adhesion (%) = (1 − A/A_0_) × 100, where A_0_ is the absorbance of the bacterial suspension before mixing and A is the absorbance of the aqueous phase after mixing. All experiments had three biological replicas, each with three technical controls.

### 2.7. Biofilm Formation Assay

The modified test tube method described by Christensen et al. (1982) was used to assess biofilm formation [26]. A 100 μL aliquot of an overnight culture of the studied strains grown in the R2A medium was inoculated in 1 mL of R2A medium supplemented with hexadecane or crude oil (final concentration 1%). The glass test tubes were incubated at 30 °C, and biofilm development was measured after 24 and 48 h. First, 250 μL of crystal violet (1%) was added to the growth medium, and after 15 min staining, all liquid was removed. The excess stain was eliminated with deionized water (washing was repeated three times). After that, 1.2 mL of ethanol was added to the dry tube to solubilize the stain absorbed by cells forming a biofilm. The absorbance of the solution was measured spectrophotometrically at 540 nm. Each assay was performed at least three times with three technical parallels.

### 2.8. Biofilm Staining and Confocal Laser Scanning Microscopy (CLSM) Imaging

Strains ICP1 and ICTN13 were grown in glass containers containing R2A medium or R2A supplemented with hexadecane (1%) at 30 °C for 24 h. The biofilm development was observed on cover glasses (18 × 18 mm) placed in the glass container. For CLSM analysis of the biofilm, the cover glasses were removed from the growth medium and washed gently with 1xME to remove free-living cells. The attached cells were stained with the LIVE/DEAD^®^ BacLightTM Bacterial Viability Kit L7012 (Invitrogen; Waltham, MA, USA) for 20 min. The 3D images were obtained with a confocal microscope (Olympus FV1000 system OVF 10278 with inverted 20× objective) and analyzed with Imaris (7.6.5; Bitplane AG; Zürich, Switzerland). At least four images were taken in different sample areas. Biofilm thickness values were calculated considering the number of CLSM sections on the z-axis (distance between each 1.2 μm section) with AutoQuantX (3.0.4; Media Cybernetics Inc.; Rockville, MD, USA).

### 2.9. Screening of Biosurfactant Production

The biosurfactant production was assayed by a modified method developed initially by Siegmund and Wagner (1991) [27]. The M9 minimal medium plates supplemented with 0.1 mg/mL cetyltrimethylammonium bromide (CTAB; Sigma-Aldrich (St. Louis, MO, USA) and 5 μg/mL methylene blue were inoculated with the ICP1 and ICTN13 strains. *Pseudomonas putida* KT2440 and *Pseudomonas aeruginosa* PAO1 strains were used as negative and positive controls, respectively. The cells were grown in hexadecane vapors for 48 h at 30 °C.

## 3. Results and Discussion

### 3.1. Role of the Pre-Growth Substrate on the Growth of the Strains on Phenol, Hexadecane, or Their Mixture

**Figure 1:**
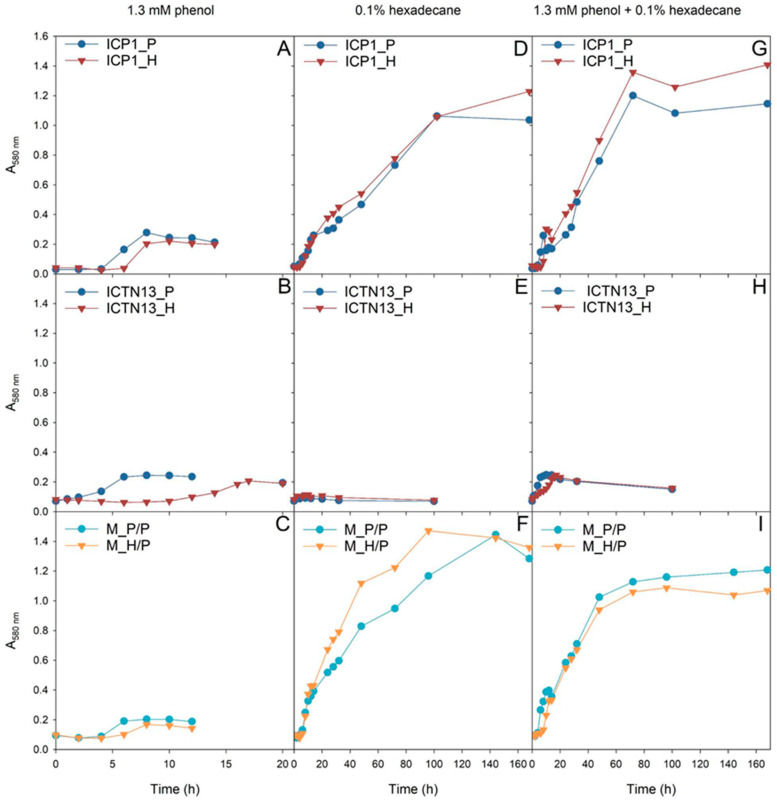
The growth of the *A. venetianus* strain ICP1 (**A**,**D**,**G**), *P. oleovorans* strain ICTN13 (**B**,**E**,**H**), and the mixture of strains (**C**,**F**,**I**) on M9 minimal medium containing 1.3 mM phenol (**A**–**C**), 0.1% hexadecane (**D**–**F**), or both substrates (**G**–**I**). The cells of the pure cultures were pre-grown on R2A medium containing phenol (P; blue) or hexadecane (H; red). In the case of the mixture of strains (**C**,**F**,**I**), the cells of strain ICTN13 were always pre-grown on phenol-containing R2A medium and strain ICP1 on phenol (P/P; light blue)- or hexadecane (H/P; orange)-containing R2A medium.

First, it was necessary to characterize the growth of the studied strains as the precise growth characteristics help to understand the behavior of bacteria in polluted environments. Two aspects are important for crude oil degradation: how quickly bacteria adapt to oil components, i.e., the length of the lag phase, and the cooperation of bacteria in the degradation of individual carbon sources. Therefore, the length of the lag phase of bacteria pre-grown in different C sources was assessed in M9 minimal medium with model substrates, phenol and hexadecane, in cultures of single strains and the mixed culture of strains (Figure 1).

The growth of the *P. oleovorans* strain ICTN13 on phenol was affected by the pre-growth conditions, as the lag-phase length was much longer if the cells were pre-grown on hexadecane-containing R2A medium (Figure 1B). Notably, the strain ICTN13 was not able to grow in liquid M9 minimal medium supplemented with hexadecane (Figure 1E). Therefore, the biomass yield in the mixture of substrates (Figure 1H) was similar to that obtained in the phenol-containing medium (Figure 1B).

In the case of the *A. venetianus* strain ICP1, the pre-growth medium moderately affected phenol degradation. However, the lag-phase length was slightly longer when cells were pre-grown on hexadecane (Figure 1A). Unlike strain ICTN13, strain ICP1 had a high biomass yield when grown on hexadecane (Figure 1D), and the effect of pre-growth conditions on lag-phase length was insignificant. Our results implied that in the mixture of substrates, the strain ICP1 first consumed phenol, and after a short second lag-phase (~10 h), hexadecane was degraded (Figure 1G). This assumption was based on the observation that during the degradation of phenol (Figure 1A), colored intermediates formed in the growth medium. This phenomenon was also observed in the mixed substrate cultivation (after ~10 h incubation) and agreed with the results obtained in our previous study [2].

The experiments with the mixture of ICP1 and ICTN13 revealed the cooperation of the strains in the degradation of the studied substrates. Although the strain ICTN13 did not utilize hexadecane, this strain supported the growth of the strain ICP1. Consequently, the growth of the mixture of strains on a hexadecane-containing medium was enhanced as we observed that the mixed culture’s optical density was increased compared with cultures of single strains (Figure 1F). For example, the biomass formation by the strain ICP1 (Figure 1D) was approximately two times smaller than the mixed culture after 48 h growth on hexadecane (Figure 1F). In the case of the mixture of substrates (Figure 1G,I), a smaller amount of colored intermediates and a shorter second lag-phase (~5 h) was observed after phenol consumption by the mixed culture in comparison with the ICP1 culture (Figure 1G).

The experiments revealed the different growth behaviors of the ICP1 and ICTN13 in phenol- and/or hexadecane-containing media. To assess the growth parameters of these strains in a more complex pollutant-containing environment, we next examined the strains’ growth ability in crude oil-supplemented medium.

### 3.2. Growth of the Strains on Crude Oil

**Figure 2:**
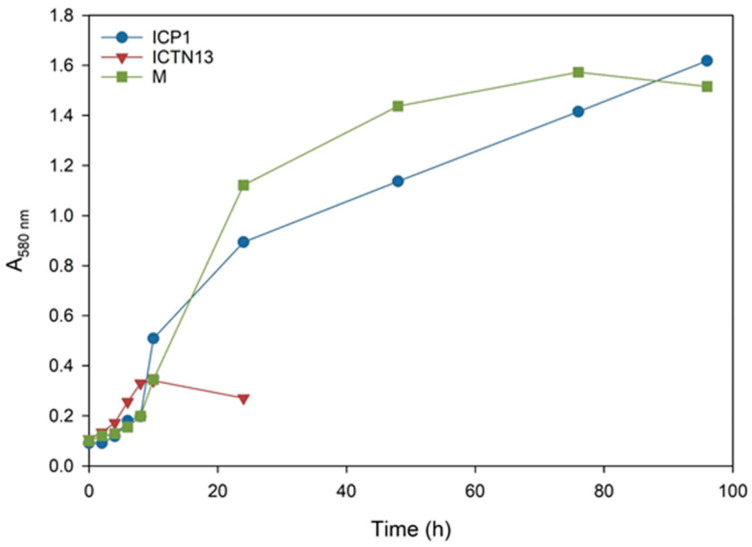
The growth curves of the *A. venetianus* strain ICP1 and *P. oleovorans* strain ICTN13 and the mixture of strains (M) cultivated on M9 minimal medium containing 1% crude oil at 30 °C.

**Figure 3:**
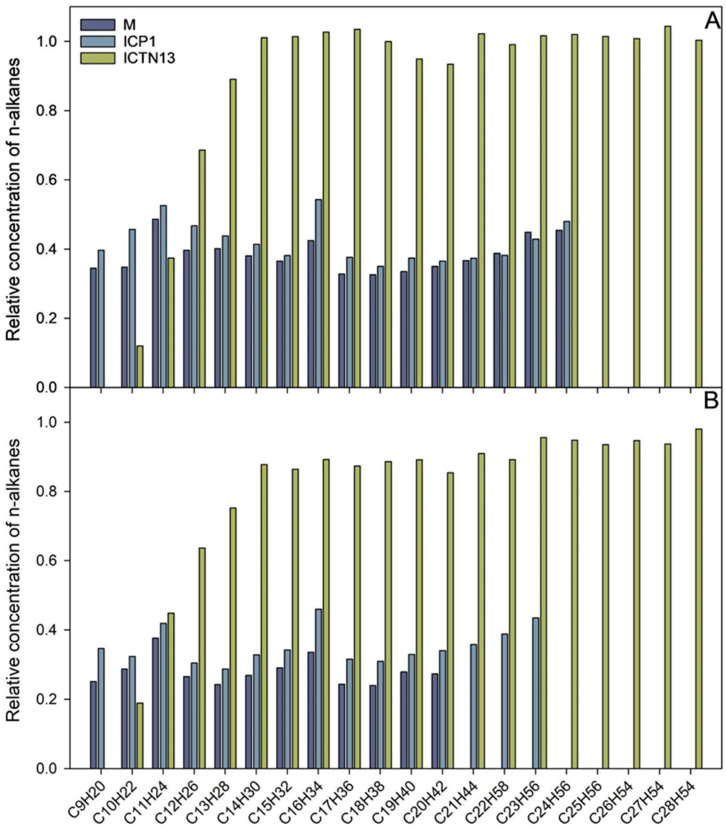
The relative concentration of n-alkanes after 24 h (**A**) and five days (**B**) incubation of *A. venetianus* strain ICP1 and *P. oleovorans* strain ICTN13 and the mixture of the strains (M) in M9 minimal medium containing 1% crude oil at 30 °C. The content of n-alkanes in the samples is expressed as the relative concentration compared to the abiotic control.

Pollution is often caused by mixtures of different compounds; for example, various aliphatic (n-alkanes, branched, and cyclic alkanes) and aromatic (low molecular weight and polyaromatic) hydrocarbons enter the environment as a result of oil spills. In the subsequent experiments, we evaluated the ability of the strains ICP1 and ICTN13 to grow (Figure 2) and utilize alkanes (Figure 3) in the M9 minimal medium supplemented with 1% crude oil.

The increase in the absorbance observed in the flasks inoculated with the strain ICTN13 (Figure 2) was probably caused by the degradation of aromatic compounds found in the crude oil; as for the aliphatic compounds, only shorter alkanes (C_9_–C_13_) were partly utilized (Figure 3).

The five-day experiment revealed that the strain ICP1 efficiently degraded aliphatic compounds (Figure 3) and produced a vigorous amount of biomass (Figure 2). During the first 24 h, this strain wholly took up alkanes with 25 to 28 carbons, and the alkanes C_9_ to C_24_ concentrations were diminished by 50–60%. After 5 days of incubation, C_24_ alkane was removed, and the concentration of shorter alkanes was reduced by 60–70% compared to initial levels.

**Figure 4:**
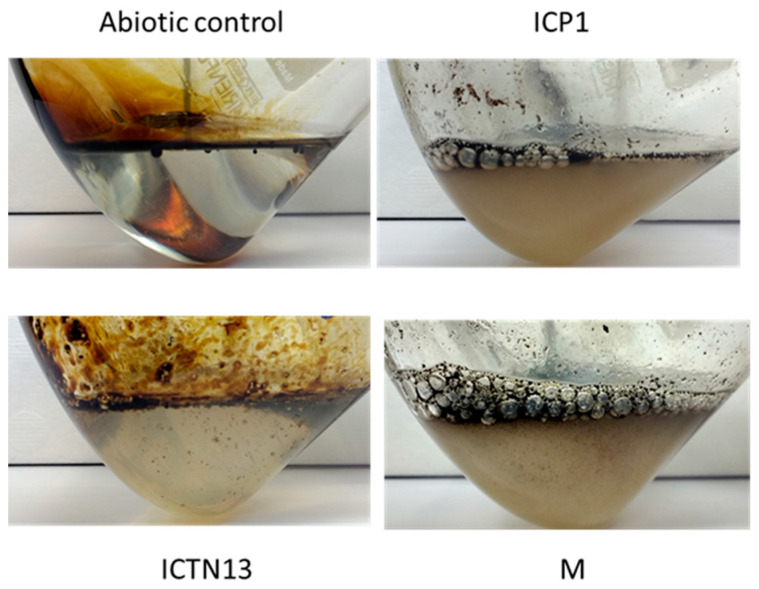
Photos of the flasks after 24 h incubation of abiotic control, the *A. venetianus* strain ICP1, the *P. oleovorans* strain ICTN13, and the mixture of strains (M) in M9 minimal medium with 1% crude oil at 30 °C.

At the same time, improved degradation of alkanes was observed with the mixture of the strains ICP1 and ICTN13, as alkanes longer than 21 carbons were utilized within 5 days (Figure 3). It is essential to mention that after 24 h of incubation, the walls of the flasks containing ICP1 alone or the mixture of the strains were clean from the oil. In contrast, the flasks of the abiotic control and ICTN13 were oily, indicating the ability of the strain ICP1 to produce hydrocarbon-dissolving substances (Figure 4 and Figure S1).

Since aliphatic hydrocarbons of the crude oil are growth substrates for the ICP1 and ICTN13 strains, the next step of the study was to investigate the genetic background behind the degradation of structurally diverse hydrocarbons.

### 3.3. Alkane Hydroxylase of the Strain ICTN13

**Figure 5:**
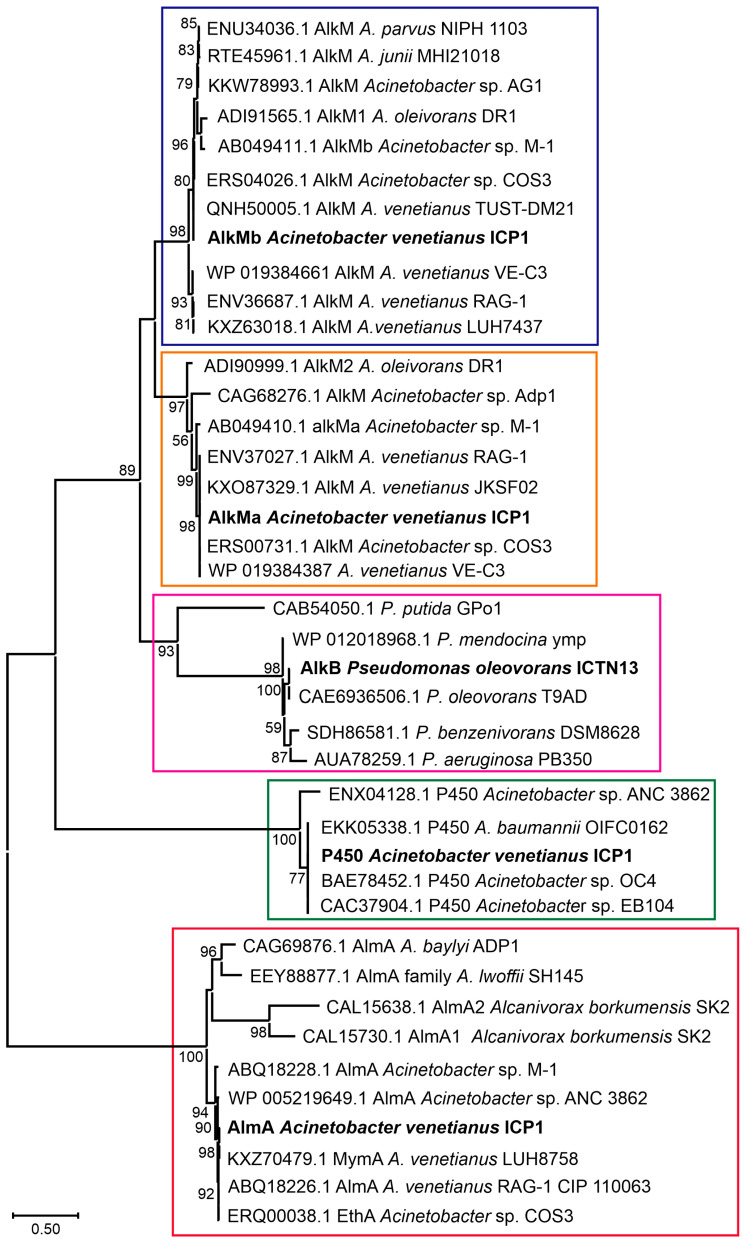
The phylogenetic analysis of the alkane hydroxylases using the Maximum Likelihood method and JTT matrix-based model. The alkane hydroxylases of *A. venetianus* ICP1 and *P. oleovorans* ICTN13 are labeled in bold text. Enzyme groups are separated with boxes (AlkMb—violet, AlkMa—orange, AlkB—cyan, P450—green, AlmA—red). Reference sequences obtained from GenBank are labeled with accession numbers and the strain’s name. The percentage of trees in which the associated taxa clustered together is shown next to the branches (>50%). The scale bar represents one substitution per amino acid. The analysis involved 40 amino acid sequences. There were 595 positions in total in the final dataset. Evolutionary analyses were conducted in MEGA X [32].

**Figure 6:**
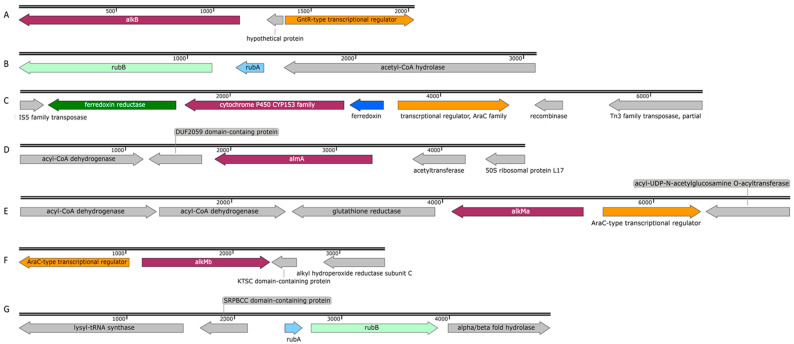
Schematic profile of the genomic organization of the alkane hydroxylases and genes related to alkane degradation in *P. oleovorans* strain ICTN13 [(**A**) *alkB* (OK245472) and (**B**) *rubAB* (OK245473)] and in *A. venetianus* strain ICP1 [(**C**) *P450* (OK143499), (**D**) *almA* (OK181059), (**E**) *alkMa* (OK181057), (**F**) *alkMb* (OK181058), and (**G**) *rubAB* (OK245471)].

Strains from the genus *Pseudomonas* are widespread and have diverse metabolic properties, among which is the ability to degrade alkanes [28,29,30,31]. Analysis of the genomic sequence of *P. oleovorans* strain ICTN13 revealed that this genome encodes one alkane hydroxylase, AlkB. The deduced amino acid sequence of AlkB is closely related (99% identity) to the *Pseudomonas* sp. THAF42 and *P. oleovorans* T9AD AlkBs. At the same time, it only has 37% identity with AlkB of *P. putida* GPo1 [28] (Figure 5). Contrary to GPo1, the *alkB* of the strain ICTN13 is located in the chromosome, and the *alk* genes are not organized in one operon (Figure 6A,B).

Proximal to the *alkB* gene, there are genes encoding proteins involved in the degradation of purines and pyrimidines, and downstream of the *alkB* is the gene for the GntR-type transcriptional regulator. The rubredoxin RubA- and rubredoxin reductase RubB-encoding genes that are involved in the electron transfer to AlkB were found in a separate contig (Figure 6B), and they were distantly related (identity < 30%) to the respective GPo1 sequences. The gene encoding the AlkB of the ICTN13 has a similar genetic context with the strains THAF42 and T9AD and *P. aeruginosa* PAO1 (AlkB1 has 68% and AlkB2 85% identity with AlkB of ICTN13) and *P. protegens* CHA0 (40% identity with AlkB of ICTN13). Smits et al., 2002, showed that AlkBs of PAO1 and CHA0 oxidize medium-chain n-alkanes [29]. At the same time, no such data were found in the literature about the AlkBs of *Pseudomonas* sp. THAF42 and *P. oleovorans* T9AD.

The experiments performed in the current study revealed that ICTN13 could grow in a liquid medium containing crude oil, and some shorter alkanes (C_9_–C_13_) are degraded. However, further study is needed to clarify the role of AlkB in crude oil degradation. Notably, as mentioned above, the length of the lag phase in a phenol-containing medium depended significantly on the pre-growth substrate, revealing the toxic or stressful effects of the hexadecane on the strain ICTN13. However, when ICTN13 and ICP1 were mixed, the growth of bacteria and the degradation of alkanes were enhanced. The strain ICTN13 can quickly degrade phenol without the accumulation of toxic intermediates, and it also has a toluene degradation operon [2]. Therefore, the presence of phenol and toluene degradation genes in ICTN13 could be responsible for the degradation of aromatic compounds from the crude oil, thereby creating the conditions for ICP1 to utilize aliphatic compounds.

### 3.4. Alkane Hydroxylases of the Strain ICP1

The members of the genus *Acinetobacter* can degrade alkanes of different lengths (from C_6_ to C_44_) and various aromatic compounds found in crude oil [33,34]. A study by Fondi et al. (2016) analyzed the genetic/genomic characteristics of six *A. venetianus* strains and listed the important genes and catabolic pathways for evaluating alkane degradation efficiency [35]. They also noted that *Acinetobacter* strains possessing diverse alkane hydroxylases and strategies for alkane uptake are good candidates for an oil spill bioremediation consortium. The growth experiments carried out with *A. venetianus* ICP1 demonstrated that hexadecane and aliphatic components (C_9_–C_28_) of the crude oil are the growth substrates for this strain. The analysis of the whole genome sequences of the strain ICP1 revealed the presence of three different types of alkane hydroxylases: cytochrome P450 of the CYP153 family, integral membrane non-heme di-iron alkane hydroxylase AlkM, and flavin-binding monooxygenase AlmA, totaling four alkane hydroxylase-encoding genes (*alkMa* and *alkMb*, *almA*, and *P450)* (Figure 5 and Figure 6C–G). All alkane hydroxylases clustered into separate clusters on the phylogenetic tree and were genus related. The genetic organization of these genes was similar to that of the previously described *Acinetobacter* strains [11,36,37,38].

Two integral-membrane non-heme di-iron alkane hydroxylase-encoding genes, *alkMa* and *alkMb*, of the strain ICP1 were identified in the same contig, but a 403,859 bp sequence separated them. The genomic organization of the *alkM* genes in ICP1 (Figure 6E,F) was the same as in *A. venetianus* strains RAG-1 [3] and VE-C3 [39], and *Acinetobacter* sp. Strains COS3 [40], M-1 [41], and DR1 [42]. ICP1 AlkMa and AlkMb amino acid sequence identities with the corresponding proteins of the reference strains were from 82 to 100% and 92 to 100%, respectively (Table S2). At the same time, the identity between the AlkMa and AlkMb sequences was considerably lower; for the strains ICP1, M-1, DR1, RAG-1, VE-C3, and COS3, it was 68, 52, 61, 66, 59, and 58%, respectively. Aligning these sequences and the AlkB sequences of the *P. putida* GPo1 revealed that in position 55, the C_6_–C_13_ alkane-degrading strain GPo1 has a tryptophan (W) residue. In contrast, *Acinetobacter* strains had leucine (L) or isoleucine (I) in this position. Such replacements have been previously associated with the ability of alkane hydroxylases to degrade alkanes longer than 13 carbon atoms [43]. The previous studies have shown that the expression of *alkMa* is induced by alkanes longer than C_22_ [3,39,40], while the expression of *alkMb* is induced in the presence of C_10_ to C_22_ alkanes [3,38,42].

The genes *rubA* and *rubB* encoding rubredoxin and rubredoxin reductase, responsible for electron transport to AlkM, were found in a separate operon in the genome of the strain ICP1, similarly to other alkane-degrading *Acinetobacter* strains [44]. The deduced amino acid sequences of the RubA and RubB of the strains ICP1 and COS3 shared 100 and 98% identity, respectively. Ratajczak et al., 1998, showed that these proteins are constitutively expressed and involved in other electron transfer reactions [44].

The 1515 bp gene encoding the alkane hydroxylase P450 is located between the genes encoding ferredoxin reductase and ferredoxin. The deduced amino acid sequences of these genes were >99% identical with those found on the plasmid pAR3 of the *A. radioresistens* DD78 (Figure 6C). Upstream of the ferredoxin gene, an AraC-type transcriptional regulator gene was identified that might be responsible for regulating the expression of *P450* (Figure 6C). In the genome of strain DD78, the genes for two transposases belonging to the IS5 and Tn3 families are located near the *P450* operon [36]. The same type of transposase-encoding genes were also found close to the *P450* operon in the genome of the strain ICP1. These data indicate the possibility of distributing the *P450* operon via mobile genetic elements. A highly similar operon was also identified on plasmid pAC450 (AJ311718.1) of the *Acinetobacter* sp. EB104 [45]. Maier et al., 2001, showed that *P450* of the strain EB104 is a soluble protein with hydrophobic amino acids responsible for the association with membranes and the contribution to the specific substrate binding and hydroxylation of unsubstituted n-alkanes [45]. The proposed substrate range of *P450* includes C_5_–C_16_ alkanes, alkylbenzenes, and alicyclic compounds [10].

The flavin-binding monooxygenase, AlmA, associated with the degradation of longer n-alkanes (C_24_–C_36_), is widely distributed among bacteria [46]. The genetic context of the AlmA-encoding genes of the strain ICP1 (Figure 6D) more closely resemble that of *A. venetianus* RAG-1 [35]. The only difference in the 6238 bp sequences appeared upstream of the *almA*: in the strain RAG-1, an ORF encoding a hypothetical protein was found, whereas, in ICP1, a putative acetyltransferase gene that is transcribed in a different direction was identified. Almost similar genetic organization of the genes was also reported in *Acinetobacter* sp. ADP1, although at both sides of the *almA*, there was one missing ORF [11]. AlmA of ICP1 appeared to be 99% and 77% identical with the AlmA sequence of RAG-1 and ADP1, respectively.

Overall, previous studies have shown that different classes of alkane hydroxylases confer the ability to degrade alkanes of specific chain lengths, while the substrate range of the enzymes may overlap [35]. Possessing multiple enzymes with the same function could confer greater efficiency in the degradation of mixtures of alkanes and an advantage to the strains to survive in a polluted environment. Therefore, the following experiments aimed to determine the mRNA amount of alkane hydroxylases encoding genes in the cells of ICP1 grown on a medium containing different n-alkanes, branched alkanes, or crude oil.

### 3.5. Transcription of Alkane Hydroxylases in the Strain ICP1

Most *Acinetobacter* strains studied so far have multiple alkane hydroxylases. For example, *A. venetianus* RAG-1, which degrades C_10_–C_38_ n-alkanes, has two homologous genes encoding AlkMa and AlkMb, and one gene for AlmA [3,35]. Experiments by Liu et al., 2021, showed that transcriptional activation of *alkM* genes was differentially induced in the presence of alkanes [3]. Namely, *alkMb* was expressed at a higher level with C_12_–C_16_ and *alkMa* with C_20_–C_32_, whereas *almA* was constitutively expressed. Therefore, we were curious whether transcriptional activation of genes encoding alkane hydroxylases in ICP1 cells depends on alkane chain length and whether they have overlapping substrate ranges.

Before measuring relative amounts of mRNA in cells, we assessed the bacterial growth. The R2A medium supplemented with 0.2% n-alkane (C_9_, C_12_, C_14_, C_16_, C_18_, C_22_, or C_32_), branched alkane (squalene, S), crude oil (CO), or without supplementations was inoculated with *A. venetianus* ICP1. The growth of bacteria was monitored spectrophotometrically at 580 nm (Figure S2). In general, it can be summarized that the added alkanes in the R2A medium positively affected the bacterial biomass formation, which means they behave as an additional C source to ICP1 (Figure S2). However, despite the supplemented alkane, bacteria grew similarly to the (mid-)exponential growth phase (A_580_~0.6), indicating that R2A contains sufficient energy and C sources to support bacterial growth. The highest biomass yield was obtained when the strain ICP1 was grown in a C_16_-containing medium (maximum A_580_ was 2.5), whereas with the crude oil, C_18_, C_14_, C_32,_ and C_22_ slightly lower values were observed (maximum A_580_ was 1.5–2.0). It is striking that the biomass yield with C_9_, C_12,_ and branched alkanes differed little from the biomass yield of cells grown in R2A without supplements (Figure S2).

**Figure 7:**
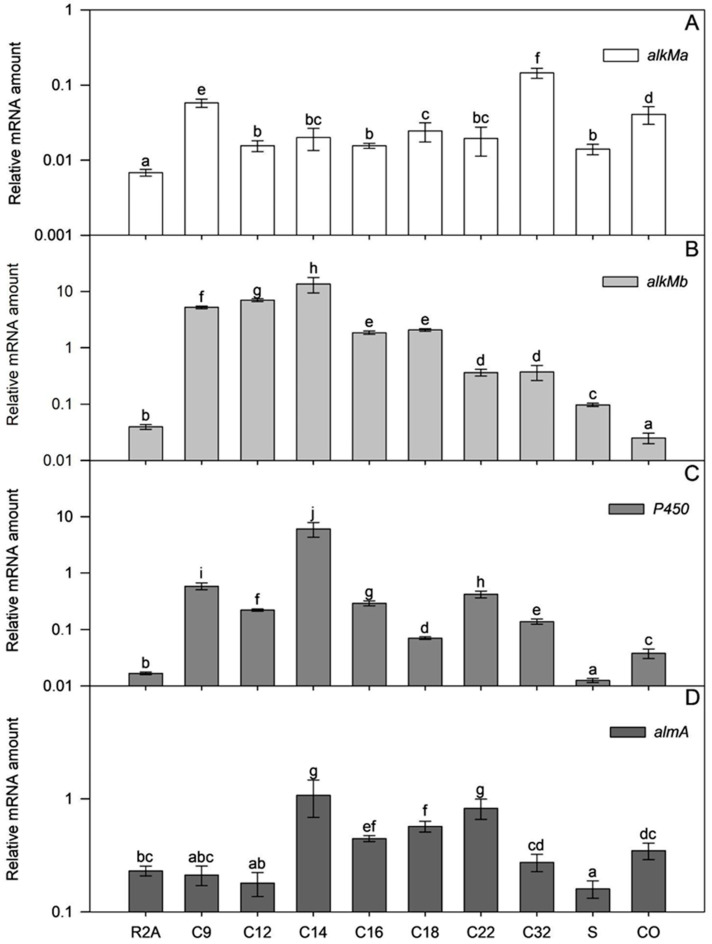
Relative transcription of *alkMa* (**A**), *alkMb* (**B**), *P450* (**C**), and *almA* (**D**) of *A. venetianus* ICP1 grown in R2A medium without and with alkanes (C_9_, C_12_, C_14_, C_16_, C_18_, C_22,_ and C_32_; squalane, S; crude oil, CO). RNA was isolated from samples taken from the exponential growth phase (Figure S2; A_580_~0.6). The amount of mRNA of the studied genes (y-axis) is expressed as relative values to the mRNA of the reference gene *rpoB*. Averages of three biological experiments with three technical parallels are presented with 95% confidence intervals. The lowercase letters show the homogenous groups, and identical letters denote non-significant differences (*p* ≥ 0.05) between the amount of relative mRNA of the gene.

The mRNA amount of four alkane hydroxylase-encoding genes was determined in exponential (A_580_~0.6) and early stationary growth phase (A_580_ ≥ 1) ICP1 cells. The mRNAs of all genes were detectable at both growth phases, including cells grown in an alkane-free medium (Table S3). These data suggest that the studied genes are always expressed to some extent, even without alkanes in the medium (Figure 7). The amount of *almA* mRNA in cells grown in the R2A medium without alkanes was 6 to 33 times more abundant than *alkM* or *P450* mRNAs. Comparing the mRNA amounts from the cells grown with alkanes to those not exposed to alkanes, in most cases, alkanes induced the transcription of the studied genes (Figure 7, Table S3). Only the transcription of *almA* seemed less sensitive to alkanes in the R2A growth medium and, was constitutively transcribed instead.

In alkane (C_12_–C_22_)- or crude oil-supplemented media, the *almA* mRNA amount in the exponential growth phase ICP1 cells increased up to 4.7 times compared to those grown in R2A without alkanes (Table S3). The flavin-binding monooxygenase, AlmA, is widespread among bacteria and is associated with the degradation of longer n-alkanes (C_24_–C_36_) [46]. However, it is also expressed in the presence of shorter alkanes, such as C_14_, in the cells of the *Alcanivorax borkumensis* [47] and branched alkane pristane in *Marinobacter* species strains [46]. In the current study, the transcriptional activation of *almA* (Figure 7, Table S3) by substrates was modest in exponential growth phase ICP1 cells. Thus, transcriptional regulation does not seem to be the most critical step for regulating *almA* expression in ICP1. Instead, it rather occurs via post-transcriptional steps or allosterically.

The mRNA amount of *alkM* genes increased significantly if alkanes were added to the R2A medium (*alkMa* increased up to 21 times and *alkMb* up to 364 times compared to bacteria grown in R2A without alkanes; Figure 7 and Table S3), indicating that alkanes can induce the transcription of *alkM*. Moreover, the amount of *alkMa* mRNA in the cells did not depend on the length of alkanes added to the medium (ρ = 0.187; *p* = 0.153; R2A, R2A+squalene, and R2A+crude oil data were excluded from the analysis).

On the other hand, the amount of *alkMb* mRNA depended on the length of the added alkane. A strong negative correlation was ascertained in amounts of *alkMb* mRNA and the length of the n-alkane used in the medium (ρ = −0.795, *p* < 1 × 10^−6^; R2A, R2A+squalene, and R2A+crude oil data were excluded from the analysis). This indicates that the transcription of *alkMb* depends on the length of the alkane in the growth medium, and transcription is an important step in regulating *alkMb* expression (Figure 7B). The same trend has been observed in previous studies with *A. oleivorans* DR1 [42] and *A. venetianus* RAG-1 [3]. Interestingly, as in the case of the DR1 [42], the relative transcription of *alkMa* in ICP1 was lower than *alkMb*. Unlike *alkMa*, crude oil did not increase the amount of *alkMb* mRNA in exponential growth phase cells. The regulation of *alkMb* expression may be more complex, and some components of crude oil could repress *alkMb* transcription or induce the fast degradation of its mRNA.

A negative correlation was obtained between the mRNA amount of *P450* and the n-alkane length (ρ = −0.488, *p* = 5 × 10^−5^; R2A, R2A+squalene, and R2A+crude oil data were excluded from the analysis) in ICP1. However, it was impossible to detect a clear correlation between the alkane length and mRNA abundance as was done for *alkMb*. The amount of *P450* mRNA was somewhat dependent on a single alkane, such as C14, when the amount of *P450* mRNA was 364 times higher than that of *P450* mRNA in R2A (Figure 7). The increase of *P450* mRNA by other used alkanes was ten or more times smaller (Figure 7), indicating that C14 could be the specific effector for *P450* transcriptional activation. Contrary to n-alkanes and crude oil, the branched alkane squalene did not increase the amount of *P450* mRNA in cells, indicating that the induction of *P450* transcription is somewhat specific to unbranched alkanes (Figure 7).

Next, we were interested in whether ICP1 explicitly uses one of the four genes to degrade alkanes and crude oil or whether all four genes are essential simultaneously. Since R2A is a relatively carbon-poor medium, the degradation of alkanes as one of the main C-sources should be substantial in stationary growth phase cells. Therefore, the relative mRNA abundance of the genes was compared in the stationary and exponential growth phase cells (Table S3). We were aware that estimating the gene’s importance via mRNA abundance is insufficient but still somewhat indicative of relevance.

The transcription of *alkMb* was relatively high or increased in stationary-phase cells in the presence of almost all studied alkanes except C_12_ and branched alkanes (Table S3), indicating that *alkMb* could be the primary gene that supports growth on media alkanes and crude oil. On the other hand, the transcription of *almA* did not markedly increase in stationary-phase cells compared to the exponentially growing cells, except with C_22_ and C_32_, when the increase was up to 3.1 times (Table S3). Bacteria usually induce genes for the degradation of secondary C sources and do not constitutively express them because it is too energetically expensive. Thus, in ICP1, *almA* could have a minor role in short alkane degradation. The induction of *almA* transcription by longer alkanes could be time-dependent as the solubility of such alkanes is very low.

The amount of *P450* mRNA was already high in exponential-phase cells but increased up to 19 times in stationary-phase cells compared to the exponential growth phase cells (Table S3). Therefore, *P450* could be essential in degrading these alkanes.

The amount of *alkMa* mRNA was low in exponentially growing cells in the presence of all alkanes. However, in the stationary-phase, when C_16_, C_18,_ or C_22_ was used in the medium, the *alkMa* mRNA increased up to 19-fold compared to the exponential growth phase cells. We cannot rule out the possibility that the post-transcriptional steps of *alkMa* are significantly effective, and for ICP1, AlkMa is essential for medium-length alkane degradation. However, both genes, *P450* and *alkMa*, still seem to play a secondary role in the degradation of alkanes and crude oil, while the primary player is *alkMb*.

Among the tested hydrocarbons, we also used the branched alkane squalane. The biomass yield increase of strain ICP1 was minimal in the medium containing squalane compared to the medium without an inducer (Figure S2). Additionally, the relative transcription of alkane hydroxylase-encoding genes was low compared to the n-alkanes (Figure 7). There are a few papers describing microorganisms that can degrade squalane (2,6,10,15,19,23-hexamethyltetracosane, C_30_H_62_), for example, *Alkanindiges illinoisensis* [48] and different *Mycobacterium* species [49]. However, more reports have been published about the degradation of branched alkanes shorter than squalane, e.g., pristane (2,6,10,14-tetramethylpentadecane; C_19_H_40_) and phytane (2,6,10,14-tetramethylhexadecane; C_20_H_42_) by the strains from the genera *Marinobacter*, *Alcanivorax*, *Nocardia*, and others. For example, in *Alcanivorax borkumensis*, SK2 homologs of *almA* and *P450* were expressed in the presence of these compounds. [47]. Branched-chain alkanes are often more difficult to degrade because their entry into cells or binding to the enzyme’s active site is disrupted by alkane side chains. The alkane structure may also limit the activity of the following β-oxidation pathway enzymes. [50]. To confirm the ability of the strain ICP1 to degrade branched chain alkanes, additional experiments with shorter branched alkanes should be performed, accompanied by the determination of gene expression and the substrate degradation rate.

Crude oil is a complex mixture that contains n-alkanes, branched alkanes, aromatics, and polycyclic aromatics. Although the composition of the crude oil varies from site to site, it mainly has C_10_–C_35_ n-alkanes [51]. The amount of mRNA expressed from the *alkMb* gene was 1063-fold higher in the stationary-phase ICP1 cells compared to the exponential-phase cells. Additionally, the relative amount of *alkMb* was the highest (26.77) compared to the other genes (0.280–0.344) in stationary-phase cells (Table S3). This indicates the importance of *alkMb* expression for crude oil degradation in ICP1. At the same time, the amount of the *alkMa* and *P450* mRNA increased 8.3- and 7.4-fold in the stationary-phase ICP1 cells, respectively, revealing again the effect of time on the induction of gene transcription. The low transcriptional activation of *almA* by crude oil may be due to the low concentration of longer alkanes.

The performed experiments revealed that alkanes differentially induce four alkane hydroxylase encoding genes in the strain ICP1. Additionally, the expression of the studied genes depends on the alkane length and structure. How exactly the expression of these genes is regulated needs more investigation because other molecular mechanisms of cell metabolism, for example, catabolite repression, fatty acid metabolism, cell hydrophobicity, etc., have also been reported [52].

### 3.6. Hydrophobicity of the Cell Surface

The cell surface properties play an essential role in utilizing hydrophobic pollutants and adhering the cells to different surfaces [53]. The cell surface of the *A. venetianus* strain RAG-1 is hydrophobic, and adhesion to the hydrophobic alkanes is controlled by fimbriae [54]. Later, Kothari et al. (2016) showed that the pilus-coding gene cluster was upregulated in C_12_-grown RAG-1 cells [55].

**Figure 8:**
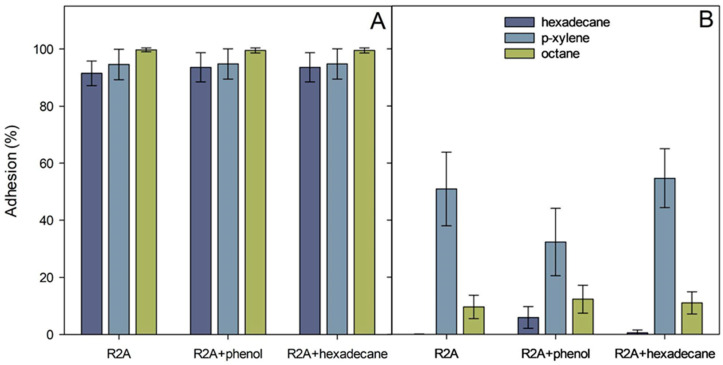
Adhesion (%) of *A. venetianus* ICP1 (**A**) and *P. oleovorans* ICTN13 (**B**) cells to hexadecane, octane, and p-xylene. Cells were grown overnight on R2A medium, R2A supplemented with 1.3 mM phenol or 0.1% hexadecane. The average values (three biological experiments with at least with three technical parallels) obtained from the MATH test are presented with standard deviations.

The relative cell surface hydrophobicity of the strains ICP1 and ICTN13, expressed here as adhesion (%) to solvents, was determined with the MATH test [25]. The calculated adhesion values to octane, hexadecane, and p-xylene are presented in Figure 8. The adhesion of the *P. oleovorans* ICTN13 to hydrophobic solvents was weak, especially with alkanes (<15%) (Figure 8B). Higher values, approximately 50%, were obtained if p-xylene was used. The adhesion was high in the case of the strain *A. venetianus* ICP1 (>91%). In contrast, the growth medium (R2A and R2A supplement with phenol or hexadecane) and the applied solvent did not affect the measured values (Figure 8A). The water layer of the MATH test performed with the strain ICP1 contained few cells compared to the hexadecane layer, as can be seen in the images taken with a microscope (Figure S3). The opposite result, the high number of cells in the water layer sample, which supports MATH test results, was obtained with the strain ICTN13 (Figure S3).

As in the case of the strain ICP1, the experiments with *A. venetianus* VE-C3 revealed that while growing in an alkane-containing medium, the bacteria form aggregates, which attach to the fuel droplets [56]. In addition, it was suggested that such droplets might incorporate into the biofilm matrix resulting in enhanced degradation of alkanes [56]. Observing hexadecane droplets in media containing strain ICP1 suggests the presence of bioactive compounds, such as surfactants, that increase the surface area and bioavailability of hydrophobic compounds.

### 3.7. Biofilm Formation Ability of the Strains in the Presence of Hexadecane and Crude Oil

The structure of the biofilms is usually complex. It is determined by the cooperation and competition between microbes in the degradation of growth substrates and environmental conditions. Forming biofilms on a water–oil interface is suggested to be one way for hydrocarbon-degrading bacteria to assimilate and degrade compounds with low water solubility.

**Figure 9:**
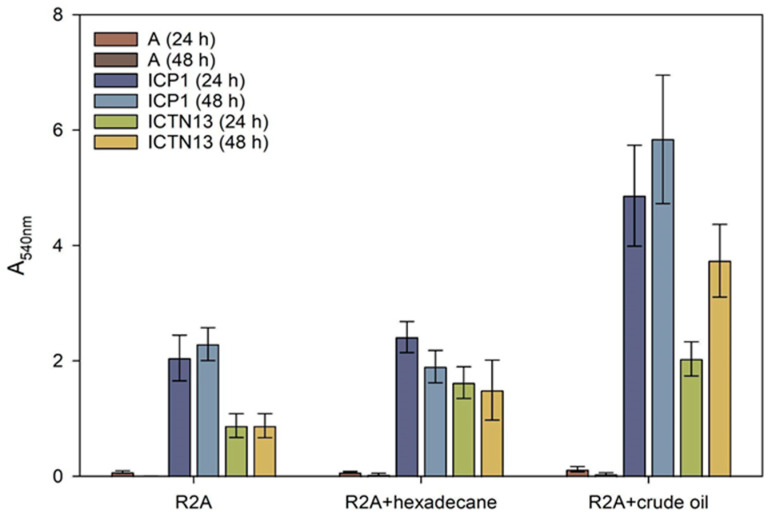
Biofilm formation on test tubes by the strains *A. venetianus* ICP1 and *P. oleovorans* ICTN13 in R2A medium and R2A medium supplemented with hexadecane or crude oil (final concentration 1%) after 24 h and 48 h incubation. Biofilm formation was measured as the absorbance at 540 nm (A_540_) of the crystal violet solution rinsed from the cells that had adhered to the walls of test tubes. The abiotic controls (without cells, designated with A) were included to show the medium’s effect in binding the stain. Error bars indicate the standard deviation of the three independent biological experiments.

An indirect method of biofilm measurement was applied to assess the amount of ICP1 and ICTN13 cells attached to glass test tubes containing R2A medium or R2A supplemented with 1% hexadecane or 1% crude oil. The results revealed that *A. venetianus* ICP1 produced up to 2.7 times more biofilms than *P. oleovorans* ICTN13 in these conditions (Figure 9). Supplementation with hexadecane or crude oil into the growth medium caused an increase in the adhesion of cells to the walls of the test tubes. Similar to the phenol-containing medium [2], the ability of the mixture of the strains to form a biofilm in hexadecane- or crude oil-containing media was lower than that of the strain ICP1 under the same conditions.

In addition, biofilm formation in static conditions to the cover glasses was studied with Confocal Laser Scanning Microscopy (CLSM) (Figure S4A–D). Cells grown at 30 °C for 24 h in R2A medium or R2A medium supplemented with hexadecane (1%) were stained with the LIVE/DEAD BacLight kit. The CLSM images showed distinguishable structures of the ICP1 and ICTN13 biofilms (Figure S4A–D). In biofilms, the cell density of strain ICP1 was higher than that of strain ICTN13. Additionally, biofilm thickness increased significantly while ICP1 was grown in a hexadecane-containing medium (Figure S4E). The number of red-stained cells was higher in the case of the strain ICTN13, which could indicate the sensitivity of cell membranes of the strain ICTN13 to hexadecane.

### 3.8. Production of Biosurfactants

Oil spill treatment is a complex task because, in addition to the differences in the composition and amount of oil, weather conditions, and environment (soil or water), the choice of optimal response methods affects the final result. One option in the marine environment is the use of dispersants that reduce the oil–water interfacial tension, as a result of which, the oil does not form thick slicks on the surface, and the formation of small oil droplets in the water column increases. Microorganisms attach to the dispersed oil droplets and degrade oil components faster than in a floating slick. However, dispersants may be toxic for marine organisms as they promote the dissolution of oil-born compounds [57]. The alternative for synthetic surface-active compounds is the use of biosurfactants [58].

**Figure 10:**
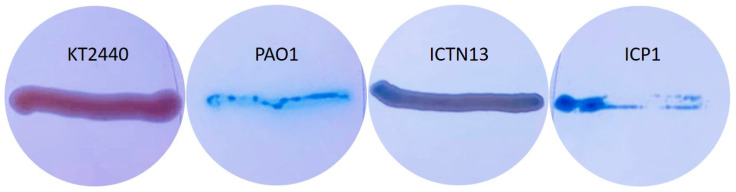
*Pseudomonas putida* KT2440 (negative control), *Pseudomonas aeruginosa* PAO1 (positive control), *P. oleovorans* ICTN13, and *A. venetianus* ICP1 grown on M9 minimal medium plates containing CTAB, methylene blue, and hexadecane (vapors).

Production and secretion of biosurfactants for enhanced degradation of hydrophobic hydrocarbons are widespread among the genera *Pseudomonas* and *Acinetobacter* strains. *Pseudomonas* strains produce glycolipids, for example, rhamnolipids, that were shown to be essential for swarming motility, involved in biofilm formation, enhancing the uptake of hydrophobic substrates like PAHs, alkanes, etc. [59]. The growth experiments in crude oil-containing media showed that strain ICP1 at least can produce compounds that dissolve oil as the flasks were not covered with oil as in abiotic control (Figure S1 and Figure 4). Therefore, we tested strains on methylene blue plates, which have been used for evaluations of the production of biosurfactants (Figure 10), and searched the genome sequences for genes relevant for their synthesis. *P. aeruginosa* PAO1 was used as a positive control (Figure 10), as it has been shown to possess genes (*rhlA*, *rhlB*, and *rhlC*) involved in the rhamnolipid synthesis, and it can produce biosurfactants at high yields [60]. The *P. putida* strain KT2440 strain did not produce rhamnolipids [59] and was used as a negative control on the methylene blue plates (Figure 10).

The analysis of the whole genome sequences of the strain ICTN13 revealed the presence of *rhlA*, which is essential for synthesizing the monorhamnolipids [59]. Although on methylene blue plates, the biomass of the ICTN13 was slightly blue, indicating surfactant production (Figure 10), additional experiments are needed to verify it.

At the same time, the biomass of the ICP1 on the same plates was as intensively blue as the positive control strain PAO1. *Acinetobacter* strains, for example, *A. venetianus* RAG-1, synthesize lipoheteropolysaccharide emulsan [61], but they can also produce different biosurfactants [62]. A 27 kbp gene cluster, which resembles an emulsan synthesis gene cluster in *A. venetianus* RAG-1 [63], was identified in the genome of ICP1. However, the structures differ, as some genes are missing or replaced. The role of this gene cluster in the production of biosurfactants will be analyzed in the future.

## 4. Conclusions

Crude oil is a complex substrate, and multiple enzymes are required for the complete degradation that in the natural environment is achieved by complementary action of different strains. The in vitro growth experiments performed with a microbial consortium composed of strains *A. venetianus* ICP1 and *P. oleovorans* ICTN13 showed enhanced crude oil degradation compared to the single strains. Further experiments are planned in the future to isolate and identify biosurfactants produced by the strains and assess their importance on alkane degradation. Improved bioavailability of the crude oil components due to the combined synthesis of the surfactants and biofilm formation could also facilitate crude oil degradation. The studies performed so far revealed that the mixture of ICP1 and ICTN13 has the potential to be used in the development of environmentally friendly and cost-effective bioremediation applications. Therefore, in situ experiments are planned to determine the survival of strains in real environmental conditions and their interactions with local microbial communities during the bioremediation of oil pollution.

## Data Availability

The isolated strains were deposited in the Collection of Environmental and Laboratory Microbial Strains (CELMS; financed by the Estonian Ministry of Education and Research (RLOMRCELMS), the public catalog of which is available on the Estonian Electronic Microbial dataBase (EEMB) website http://eemb.ut.ee (accessed on 8 February 2023)).

## Supplementary Materials

The following supporting information can be downloaded at: https://www.mdpi.com/article/10.3390/microorganisms11030752/s1, Figure S1: Flasks containing M9 minimal medium supplemented with 1% crude oil without cells (abiotic control), inoculated with *A. venetianus* strain ICP1, the *P. oleovorans* strain ICTN13, or the mixture of these strains (M) for 0 h, 24 h, and 5 days at 30 °C; Figure S2: Growth of the *A. venetianus* ICP1 on R2A medium without and with the inductor (A– C_9_, C_12_, squalane (S); B– C_14_, C_16_, C_18_; C– C_22_ and C_32_; D– crude oil (CO)). Sampling points for the isolation of RNA are marked with blue squares (A_580_~0.6; all substrates) and red circles (A_580_ ≥1; all substrates except R2A and C_9_-containing media). In the case of the crude oil second sample was taken at A_580_ = 2.3. The experiments were performed in triplicate, and representative growth curves of one experiment are shown; Figure S3: Water and hexadecane layers were sampled from the MATH test performed with *A. venetianus* ICP1 and *P. oleovorans* ICTN13 cells grown on R2A medium. Images were taken with a phase contrast microscope Olympus BX41. Scale bar, 20 µm. Figure S4. CLSM images of LIVE/DEAD stained *A. venetianus* ICP1 (A,C) and *P. oleovorans* ICTN13 (B,D) grown for 24 h on cover glasses placed into the R2A medium (A,B) or R2A medium supplemented with 1% hexadecane (C,D). The left side of each panel is a side view (x,z-plane; scale bar 20/40 µm), and the right side is a top view (x,y-plane; scale bar 20 µm) from the biofilm. The thickness of the biofilm (µm) formed on cover glasses (E). The average values of at least nine measurements are presented with standard deviation; Table S1: Primers used in this study; Table S2: Amino acid (aa) percent identity matrix for alkane hydroxylase AlkMa and AlkMb for *A. venetianus* strains ICP1, RAG-1, and VE-C3 and *Acinetobacter* sp. strains COS3, M-1, and DR1; Table S3. Relative transcription of *alkMa* (A), *alkMb* (B), *P450* (C), and *almA* (D) of *A. venetianus* ICP1 grown in R2A medium without and with the alkanes (C_9_, C_12_, C_14_, C_16_, C_18_, C_22,_ and C_32_; squalane, S; crude oil, CO). RNA was isolated from samples taken from the exponential (A_580_~0.6) and stationary (A_580_ ≥ 1) growth phases. The amount of mRNA of the studied genes is expressed as relative values to the mRNA of the reference gene *rpoB*. Averages of three biological experiments with three technical parallels are presented with 95% confidence intervals.
